# How is housing insecurity measured among older adults? A systematic review

**DOI:** 10.1093/geront/gnag130

**Published:** 2026-06-12

**Authors:** Tianxin Cai, Peng Cheng, Sin Yu Lam, Peiyi Lu

**Affiliations:** Department of Social Work and Social Administration, The University of Hong Kong, Hong Kong SAR, China; Sau Po Centre on Ageing, The University of Hong Kong, Hong Kong SAR, China; School of Political Science and Public Administration, Wuhan University, Wuhan, China; Department of Social Work and Social Administration, The University of Hong Kong, Hong Kong SAR, China; Department of Social Work and Social Administration, The University of Hong Kong, Hong Kong SAR, China; Sau Po Centre on Ageing, The University of Hong Kong, Hong Kong SAR, China

**Keywords:** Housing insecurity measurement, Systematic review, Psychometric properties, Age-friendly housing

## Abstract

**Background and Objectives:**

Housing insecurity is an established social determinant of health, with older adults being disproportionately vulnerable. Despite growing research attention, no studies have reviewed the measurement tools existing specifically for this population. This systematic review synthesizes quantitative measures of housing insecurity used among older adults, examines their dimensional structures, and evaluates their psychometric properties.

**Research Design and Methods:**

Following PRISMA 2020 guidelines, a systematic search was conducted across 5 electronic databases with no date restrictions. Eligible studies were required to include samples of older adults or mixed-age adults without an upper age limit, employ quantitative housing insecurity measures, and report psychometric evaluation.

**Results:**

A total of 1,310 articles were retrieved initially, and 13 studies were included in the final review. Most studies constructed composite indices from secondary survey data rather than purpose-built instruments. Six core dimensions were identified: housing unaffordability, instability, poor physical quality, inadequacy, lack or disrepair of housing durables, and poor neighborhood quality. Age-friendly housing characteristics were incorporated in only one study. Internal consistency was reported in two studies and test-retest reliability in none, with validity evidence largely confined to informal content justifications.

**Discussion and Implications:**

The reviewed tools showed considerable fragmentation in measurement approaches, uneven dimensional coverage, and insufficient psychometric reporting. The near-universal absence of age-friendly indicators across reviewed tools presents a notable gap. Future research may benefit from developing purpose-built, comprehensively validated instruments that integrate aging-specific dimensions across diverse policy and cultural contexts.

Housing is a fundamental human need and a critical social determinant of health (World Health Organization [WHO], 2018). Safe, stable, and adequate housing protects residents from environmental hazards and is closely associated with physical health, psychological well-being, and social functioning (Rolfe et al., 2020). In contrast, housing insecurity, encompassing poor living conditions, threatened residential stability, insufficient affordability, and hazardous neighborhood environments, has emerged as an increasingly urgent global public health concern (Cox et al., 2019; Routhier, 2019).

Against the backdrop of rapid population aging, older adults have become a particularly vulnerable group with respect to housing insecurity. Reduced income and increased time spent at home following retirement, and progressive functional limitations render older adults far more dependent on their living environments than other age groups (Cheng et al., 2025; Evans et al., 2002). Rising housing costs and limited availability of accessible and public housing have further constrained older adults’ ability to secure appropriate accommodation (Department of Housing and Urban Development [HUD], 2018; Jenkins Morales & Robert, 2020). In the United States (US), the number of older adults experiencing homelessness increased by 69% over the past decade, while coverage of federal housing assistance remains severely inadequate, with only approximately one-third of eligible low-income older households receiving support (HUD, 2017, 2018). The consequences of housing insecurity for older adults are profound (Evans et al., 2008), affecting mental health and functional independence, and are associated with increased emergency department utilization, higher rates of nursing home admission, and shortened life expectancy (Paredes et al., 2024; Rollings et al., 2022; Stahre et al., 2015).

Given its growing relevance to health, research has intensified efforts to develop housing insecurity measures. The existing operationalization mainly focuses on the adult population and has evolved from single extreme indicators to multidimensional frameworks. Early work often equated housing insecurity with homelessness or severe material deprivation, leaving many individuals situated at intermediate points along the housing insecurity continuum to fall outside the scope of research and intervention (Vásquez-Vera et al., 2017). As the field develops, housing insecurity has increasingly been defined and operationalized as a multidimensional measurement. For example, Cox et al. defined it as “inability to acquire safe, stable, adequate, and affordable housing and neighborhoods in socially acceptable ways” (Cox et al., 2019). They further proposed a seven-dimensional framework, including housing stability, affordability, housing quality and safety, neighborhood quality and safety, and homelessness (Cox et al., 2017). Other research suggested more compact approaches, such as a composite index (Routhier, 2019) or a four-dimensional tool (Gulyani & Bassett, 2010; Kantz et al., 2023). Using the 2019 American Housing Survey, researchers also developed a psychometrically validated index for large‑scale governmental use (Murdoch et al., 2022).

However, no comparable instrument exists specifically for older adult populations. For older adults, housing needs extend beyond these universal frameworks to reflect particularities of aging. As physical and cognitive capacity decline, the need for accessible features, age-appropriate housing modifications, and in-home supportive services becomes increasingly salient (Wahl et al., 2012; World Health Organization, 2018). The growing importance of these elements has been recognized by the WHO, whose Housing and Health Guidelines (2018) emphasize age-appropriate housing for individuals with functional impairments (WHO, 2018). The guidelines further advocate for integrating housing improvement into broader public health action frameworks, aiming to make cities and human settlements inclusive, safe, resilient, and sustainable.

Environmental gerontology provides a broad theoretical lens for conceptualizing housing insecurity among older adults and a foundational rationale for why this population requires a measurement framework distinct from those designed for the general adult population (Wahl & Oswald, 2016; Wahl et al., 2012). The perspective holds that as individuals age, their interaction with the residential environment changes systematically, making housing a core determinant of functional independence and health in ways qualitatively distinct from other age groups (Evans et al., 2002; Oswald et al., 2011). Accordingly, housing insecurity measurement for older adults may benefit from extending beyond physical structural quality and economic affordability to encompass neighborhood quality and community accessibility, as older adults’ daily lives are highly concentrated within the home and neighborhood environment, and these dimensions collectively contribute to the full picture of the residential environment (Padeiro et al., 2022; Wahl et al., 2012).

Within this broader framework, Person-Environment Fit theory (Lawton & Nahemow, 1973) provides more precise conceptual guidance for housing insecurity measurement, informing the selection of dimensions, the weighting of dimensions, and the content of indicators. The theory holds that functional outcomes depend on the dynamic match between personal competence and environmental press, with lower-competence individuals being disproportionately dependent on environmental support, a principle known as the environmental docility hypothesis (Park et al., 2017; Wahl & Gerstorf, 2020). Because housing deficiencies tend to produce greater impacts as health declines in older age, the dimensional structure of general-purpose tools may be insufficient to reflect the conceptual scope of older adults’ housing environment, and a dedicated measurement framework may need to advance on three fronts. First, when selecting dimensions, housing characteristics that directly affect the competence-press in older age match merit particular consideration. This provides a conceptual basis for age-friendly physical features (e.g., accessible pathways, grab bars, and fall prevention modifications) as an independent dimension (Iwarsson & Ståhl, 2003; Wahl et al., 2012). Second, when weighting dimensions, age-friendly features tend to carry greater importance for functionally impaired older adults, as small increases in environmental press may produce disproportionately large effects on lower-competence individuals. Therefore, dedicated measurement tools may assign greater conceptual weight to this dimension than general adult instruments (Park et al., 2017). Third, when determining indicator content, measurement may incorporate older adults’ subjective assessments of the person-environment fit, as this subjective appraisal serves as an important mediating mechanism through which housing affects health (Chaudhury & Oswald, 2019; Wahl & Gerstorf, 2020).

Accurate measurement of housing insecurity is essential for conducting rigorous research, evaluating interventions, and informing evidence-based policy. Adequately validated standardized tools enable the identification of high-risk populations, support the efficient allocation of housing assistance resources, and facilitate population-level tracking of policy goals (Boateng & Adams, 2023; World Health Organization, 2018). However, in contrast to fields such as food insecurity and water insecurity, where mature validated measurement systems have been established (Coates et al., 2007; Young et al., 2019), the standardization of housing insecurity measurement has lagged considerably.

Three specific research gaps are evident. First, no systematic review has synthesized the housing insecurity measurement tools used among older adults; existing reviews have focused either on general adult populations or on empirical evidence linking housing to health, without systematic attention to the distinctive circumstances of aging (Burgard et al., 2012; Leopold et al., 2016; Rolfe et al., 2020). Second, the dimensional structures and factor compositions of housing insecurity measures have not been systematically examined across the literature. Prior investigations might vary considerably in their delineation of dimensions and selection of indicators, requiring meaningful cross-study synthesis (Cox et al., 2017; Routhier, 2019). Third, the core psychometric properties of available tools, including internal consistency, test-retest reliability, and structural validity, have not been systematically evaluated across existing tools, despite their centrality to assessing measurement quality (Ayalon et al., 2019; Boateng & Adams, 2023). Drawing on these research gaps, this systematic review aims to answer three questions:

1. What tools have been used to assess housing insecurity among older adults in existing literature?
2. What dimension structures are captured by these tools?
3. What are the psychometric properties of these tools with respect to reliability and validity?

## Method

### Search strategy

This systematic review was conducted in accordance with the Preferred Reporting Items for Systematic Reviews (PRISMA 2020) checklist, following a protocol pre-registered on PROSPERO. A systematic search was conducted across five electronic databases: PsycINFO, PubMed, Web of Science, Sociological Abstracts, and Scopus. These databases were selected for their complementary coverage of the psychological, medical, interdisciplinary, sociological, and public health literature relevant to housing insecurity measurement research. Searching across multiple databases to ensure comprehensive disciplinary coverage is consistent with established practice in systematic reviews of measurement tools (Manchha et al., 2021; Peng et al., 2026). Given the anticipated limited volume of eligible studies, no date restrictions were applied.

The search strategy combined three conceptually distinct keyword groups using the Boolean operator “AND”: Group 1 (housing insecurity-related terms) AND Group 2 (measurement-related terms) AND Group 3 (older adult-related terms). The inclusion of Group 2 measurement-related terms was designed to focus retrieval on studies that involved the development or evaluation of measurement tools, rather than studies that used housing-related variables merely as exposures or covariates. This three-block search structure has been applied in comparable systematic reviews of measurement tools in adjacent fields (Hall et al., 2024; Manchha et al., 2021). Database-specific subject headings (e.g., MeSH terms in PubMed) were incorporated where applicable. The full list of search terms is presented in Table 1, and complete database-specific search strings are available in Supplementary Table S1.

**Table 1:** Search terms used in the systematic search.

Key words	No.	Search terms
Housing insecurity	1	(“housing insecurit*” OR “housing instabilit*” OR “residential insecurit*” OR “residential instabilit*” OR “housing povert*” OR “housing vulnerab*” OR “housing affordab*” OR “housing cost burden*” OR “rent burden*” OR “housing tenure” OR “housing precarit*” OR “residential precarit*” OR “housing stress” OR “housing quality”)
Measurement	2	(measure* OR scale* OR instrument* OR assessment* OR indicator* OR questionnaire* OR tool* OR index OR survey* OR validat* OR reliab* OR adapt* OR psychometric*)
Older adult	3	(aged OR elder* OR senior* OR “older adult*” OR “older person*” OR “older people” OR ag? ing OR “old age” OR geriatric* OR gerontolog* OR “late life”)

*Note.*

* denotes a truncation wildcard that retrieves all words sharing the same root (e.g., “elder*” captures “elderly,” “elders”). ? denotes a single-character wildcard that substitutes for exactly one character (e.g., “ag? ing” captures both “aging” and “ageing”).

**Figure 1:**
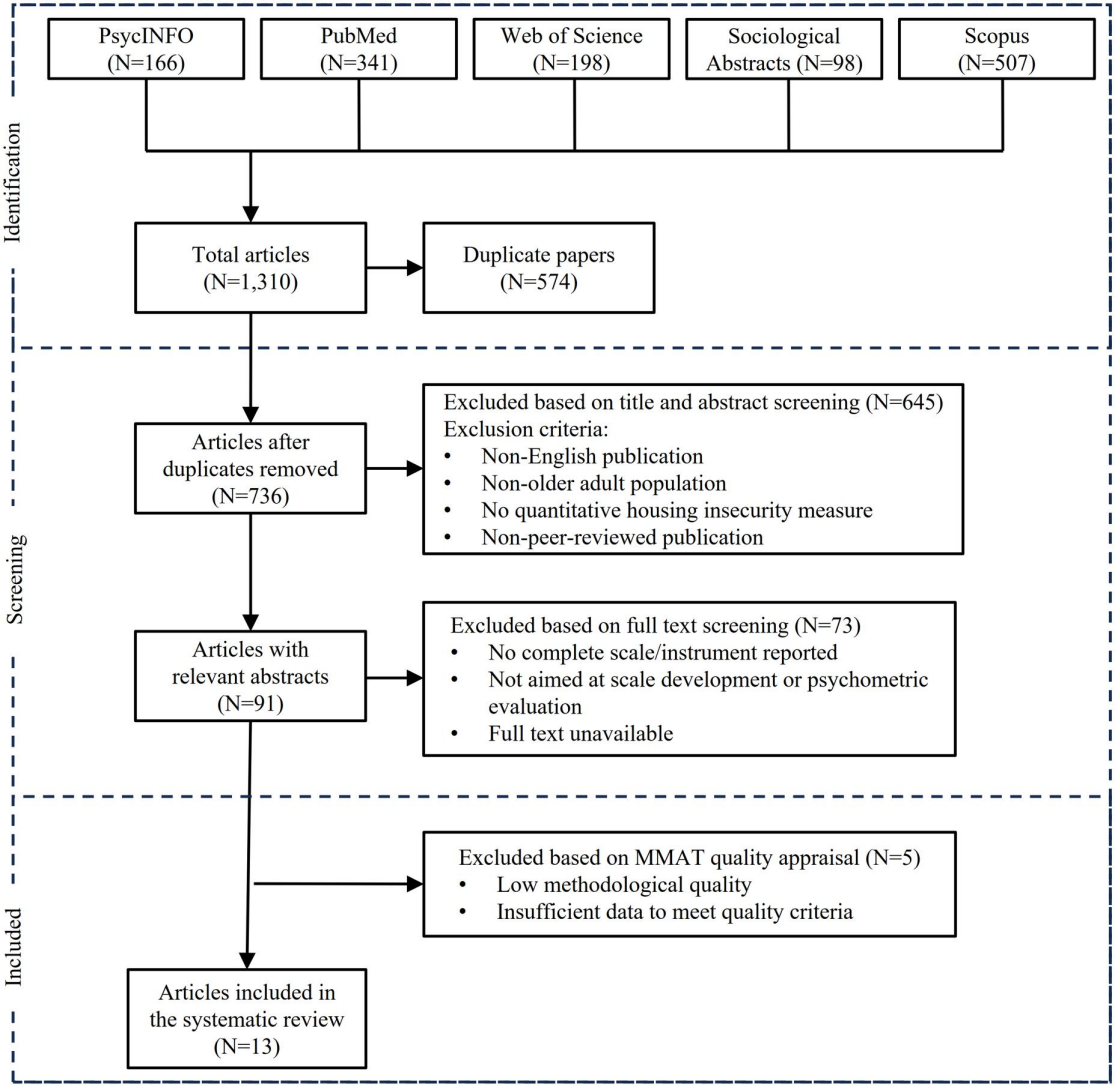
PRISMA flow diagram depicting results of search, screening, and selection processes.

Search results were first imported into EndNote X9 for initial reference management and subsequently uploaded to Covidence (Veritas Health Innovation, Melbourne, Australia) for automatic deduplication. Two independent reviewers with solid training backgrounds in social science and public health research then conducted a two-stage screening process: (1) title and abstract screening, and (2) full-text review, with reasons for exclusion documented at the second stage. Discrepancies at each stage were resolved through discussion and consensus, with a third reviewer serving as arbitrator where needed. Data were subsequently extracted by one reviewer and verified by a second. The quality of included studies was appraised using the Mixed-Methods Appraisal Tool (MMAT). The complete flow of records is documented in the PRISMA flow diagram (Figure 1).

### Inclusion criteria

Studies were considered eligible for inclusion if they met all of the following criteria: (a) it was an empirical study and its sample/population either specifically targeted older adults (aged 60 years or above) or included mixed-age samples that incorporated older participants, such as studies involving adults aged 18 and above without an upper age limit; (b) a quantitative measure of housing insecurity was used; (c) it validated or evaluated the psychometric properties of a housing insecurity tool; and (d) it was written in English and published in a peer-reviewed journal.

Studies were excluded if they focused on nonolder adult populations (e.g., studies with samples explicitly restricted to children, adolescents, or young adults with an upper age boundary below 60), lacked a quantitative housing insecurity measure (e.g., only mentioned housing insecurity conceptually or focused solely on homelessness without employing objective measurements), were not available in English, or were not published in peer-reviewed journals. The last criterion excluded conference abstracts, editorials, commentaries, review articles, and gray literature such as unpublished manuscripts, dissertations, theses, and government/organization reports. Gray literature was excluded to ensure methodological consistency and reproducibility of the review, as gray literature varies considerably in its reporting standards and peer review processes. Furthermore, detailed methodological information on measurement tools is more systematically and transparently documented in peer-reviewed publications than in policy documents or government reports.

### Data extraction

Data were extracted and coded by the first author and independently verified by a second reviewer, with discrepancies resolved through discussion and consensus. Two categories of information were extracted from each included study. The first concerned study characteristics, including author(s) and publication year, sample size, age range of participants, geographic region, study design (primary data collection or secondary data analysis), data source, number of scale items, reporter type (self-reported or observer-rated), rating scale format, and the dimensions and subscales captured by the measure. The second concerned psychometric properties, including internal consistency (Cronbach’s *α*), other reliability indicators (e.g., interobserver reliability), whether validity was reported, and the type and supporting evidence of validity assessed (e.g., content validity, construct validity). Where relevant information was not explicitly reported in a study, it was recorded as “N/R” (not reported).

### Quality appraisal

The methodological quality of all included studies was assessed using the Mixed Methods Appraisal Tool (MMAT, version 2018), a critical tool developed for the appraisal stage of systematic reviews that encompass diverse study designs, including qualitative, quantitative, and mixed methods studies (Hong et al., 2018; Pluye et al., 2009). The MMAT was selected because the included studies varied considerably in their research designs, encompassing quantitative descriptive and nonrandomized designs across both primary data collection and secondary survey analysis. Its capacity to appraise methodological quality across diverse study designs aligns well with this heterogeneity and has been applied in systematic reviews with similarly varied designs in prior research (Anantharaman et al., 2026; Zhou et al., 2022). Furthermore, as this review aimed to synthesize the breadth of existing housing insecurity measurement practices rather than to conduct an in-depth psychometric evaluation of individual instruments, MMAT’s focus on overall research design quality is more consistent with these objectives than measurement-specific appraisal frameworks. As all included studies were quantitative in design, the MMAT criteria for quantitative nonrandomized studies and quantitative descriptive studies were applied accordingly. Each criterion was rated as “Yes,” “No,” or “Can’t tell,” where the latter indicated insufficient or unclear reporting. Quality appraisal was conducted independently by two reviewers, with discrepancies resolved through discussion and consensus.

## Results

### Characteristics of included studies

Figure 1 presents the search and screening process. The systematic search retrieved 1,310 records across five databases; 736 remained after deduplication. Following title and abstract screening, 645 records were excluded, and full-text review of the remaining records yielded 13 eligible studies, all of which passed MMAT quality appraisal. Characteristics of the included studies are summarized in Table 2.

**Table 2:** Measures used to assess housing insecurity among older adults.

Article authors	Sample size	Age (years)	Region	Study	Data source	No. of items	Reporter	Rating scales	Composite/subscales	MMAT appraisal
Bhat et al. (2022)	2,598	Midlife and older adults	United States	National Study of Midlife in the United States	Secondary	5	Self-reported	Dichotomous (Y/N)	*Housing insecurity (5)*: sum up if moved in with family/friends to save money; missed mortgage or rent payment; threatened with foreclosure/eviction; lost home (foreclosure or other); experienced homelessness	*High* (5/5). Longitudinal design. National probability samples. Attrition analysis documented. Measurements and confounders addressed. Housing insecurity items consistent across waves.
Caffaro et al. (2016)	285	25-65 adults, *M* = 42.81, *SD* = 12.73	Italy	A cross-sectional study	Primary	11	Self-reported	7-point-Likert (1 = not at all functional, 7 = extremely functional)	*Indoor environment and architectural design (8)*: rooms’ area, rooms’ layout, ventilation, home exposure, summer microclimate, winter microclimate, maintenance, accessibility *Outdoor stressors (3)*: outdoor air quality, noise from adjacent houses/apartments, noise from street	*Moderate-High* (4/5). Appropriate sampling strategy, measurements, and analyses. CFA supported a bidimensional structure. Nonresponse bias unclear due to snowball recruitment and unreported response rate.
Canterberry et al. (2022)	56,155	65+, *M* = 74.0, *SD* = 5.8	United States	Health-Related Social Needs Survey	Secondary	3	Self-reported	Dichotomous (Y/N); multiple choice; 3-point scale (Y, N, already shutoff)	*Housing insecurity (1)*: living situation stability *Poor housing quality (1)*: if there are problems with pests, mold, lead paint/pipes, lack of heat, nonfunctioning oven/stove, missing/nonfunctioning smoke detectors, water leaks *Utility insecurity (1)*: utility shutoff	*Moderate* (3/5). Complete outcome data. Confounders controlled. Representativeness uncertain (24.5% response rate). Temporal inconsistency limits causal inference.
Cheng et al. (2025)	30,632	60+, median = 67 (IQR: 63, 73)	India	Longitudinal Ageing Study in India	Secondary	9	Self-reported	Dichotomous (Y/N)	*Housing quality (4)*: sum up if the house has temporary housing materials, sanitation facilities, water access, cooking fuel type, electricity availability *Related housing environmental factors (5)*: sum up if the house lacks separate bedroom, lack separate kitchen, have indoor air pollutants, household dampness	*Moderate-High* (4/5). Large nationally representative samples. Measurements and confounders addressed. Cross-sectional design limits temporal ordering.
Evans et al. (2000) ^a^	207	Women who had at least one child living in home	United States	A cross-sectional study	Primary	45	Observer	3-point-Likert (e.g., 0 = potentially dangerous, 1 = structurally sound but cracked, 2 = in good condition); dichotomous (Y/N)	*Structural quality*: e.g., ceiling/wall surface in the room *Privacy*: e.g., walk through the bedroom to get to another room) *Indoor climatic conditions*: e.g., heat has broken down *Hazards*: e.g., stairs are dangerous *Cleanliness*: e.g., clutter is in the kitchen *Child resources*: e.g., toys are accessible to children *Neighborhood quality*: e.g., houses in the immediate neighborhood had structural damage	*Moderate-High* (4/5). Two independent samples described. Psychometrics assessed (interobserver reliability, factor analysis). Longitudinal sample small (*N* = 31). Limit to low-income women.
Golant and LaGreca (1994)	12,859	60+	United States	American Housing Survey	Secondary	3	Self-reported	3-level categorical; numerical measures: 6-level categories	*Housing quality (3)*: the extent the building suffered from multiple problems (adequate, moderately deficient, severely deficient); count of physical deficiencies as many as 26; 6-level categorization of building deficiencies	*Moderate-High* (4/5). Large nationally representative AHS sample. Three valid housing quality measures used. Nonresponse bias unclear due to unreported response rate.
Jones-Rounds et al. (2014)	5,605	18-64, *M* = 40.35	Europe	WHO Large Analysis and Review of European Housing and Health Status	Secondary	N/R	Observer + self-reported	5-point-Likert for housing quality variables; various scoring for neighborhood quality variables	*Housing quality*: sum up the extent of disrepair symptoms in ceilings, floors, walls (outside and inside), doors, windows in kitchen, bathroom, corridor, and bedroom. *Neighborhood quality*: self-rate neighborhood, area rating, view satisfaction, city connections, parking, litter, vibrations, noise, recreational spaces, playground encouragement, relaxation places, safety); Observers rate green spaces, vegetation, graffiti, litter	*Moderate* (3/5). Representative multi-city sample. Appropriate multilevel modelling. Missing income data. Response rate unreported. Cross-sectional design limits temporal inference.
Kantz et al. (2023)	10,858	62-87, *M* = 72.0, *SD* = 7.6	United States	Survey of Income and Program Participation	Secondary	9	Self-reported	Dichotomous (Y/N)	*Housing quality (5)*: holes/cracks in ceiling or walls, holes in floor, plumbing problems, pest problems, household size *Housing affordability (2)*: missed housing payment, unable to pay utilities at any time in the past year *Housing stability (1)*: number of moves in past year *Neighborhood safety (2)*: unsafe neighborhood from crime, stayed home due to safety concerns	*High* (5/5). Nationally representative longitudinal SIPP data. Measurements and confounders addressed. Exposure consistent across waves .
Kim and Burgard (2022)	255	19-64, *M* = 39.5, *SD* = 1.2	United States	Michigan Recession and Recovery Study	Primary	14	Self-reported	Dichotomous (Y/N)	*Housing instability (1)*: any of the events including homelessness, eviction, moving in with others, frequent moves (>2 moves), cost-related move *Housing quality (1)*: sum up the substandard conditions including leaky roof or ceiling, plumbing-related problems, pests issues, broken windows, malfunctioning heating system/stove/refrigerator, electrical problems, peeling paint, other problems *Neighborhood (1)*: reside in a high poverty census tract (>20% families below poverty line)	*High* (5/5). Stratified random sample. High retention (>90%). Confounders addressed in statistical modelling. Measurements consistent across waves.
Nolan and Winston (2011)	2,879	65+	Ireland	EU Survey on Income and Living Conditions	Secondary	31	Self-reported	Dichotomous (Y/N)	*Housing quality (1)*: sum up if the household had bath/shower, central heating, hot water, running water, toilet, leaking roof/damp walls/rot, dark rooms *Household durables (1)*: sum up if the household had 19 items including fridge, fridge freezer, deep freeze, washing machine, clothes dryer, deep fat fryer, dishwasher, food processor, microwave, liquidiser, satellite dish, stereo, CD player, fixed line telephone, color television, vacuum cleaner, video recorder, camcorder, computer *Housing affordability (1)*: sum up if there is arrears for rent/mortgage payments, housing costs considered a heavy burden *Neighborhood condition (1)*: sum up if there is crime/violence/vandalism, noise from neighbors or street, pollution/grime/environmental problems	*Moderate-High* (4/5). Nationally representative EU-SILC data. Demographics verified against national statistics. Measurements and analyses appropriate. Nonresponse bias unclear.
Paredes et al. (2024)	6,466	65+, *M* = 77.3, *SD* = 7.7	United States	National Health and Aging Trends Study	Secondary	15	Self-reported + observers	3-level categorical for affordability; dichotomous (Y/N) for housing/neighborhood quality	*Poor housing affordability (1)*: cost burden (no: <30% of monthly income; moderate: 30%–50%; severe burden:≥50% *Poor housing quality (10):* five interior conditions (peeling/flaking paint, pests, broken furniture/lamps, flooring in need of repair, other tripping hazards); five exterior conditions (broken/boarded windows, crumbling foundation/open holes, missing bricks/siding, roof problems, uneven walking surfaces/broken steps) *Poor neighborhood quality (4):* Litter/broken glass/trash, graffiti on buildings/walls, vacant or deserted houses/storefronts, houses with foreclosure signs	*High* (5/5). National probability samples with weights. Nonresponse bias documented. Measurements based on HUD definitions. Statistical analyses appropriate.
Rollings et al. (2022)	87,348,604	18-99, *M* = 58, *SD* = 20	United States	A cross-sectional study on National Inpatient Sample	Secondary	5	Self-reported	Dichotomous (Y/N)	*Housing instability (5)*: based on *5 International Classification of Diseases, 10th Revision, Social Determinants of Health Z-Codes*, if experienced homelessness; inadequate housing; discord with neighbors, lodgers, and landlords; problems related to living in a residential institution; other problems relating to housing and economic circumstances	*Moderate* (3/5). Nationally representative inpatient data. Outcomes complete. ICD-10 Z-code likely underidentify cases. No adjustment for confounders.
Trivedi et al. (2025)	56,438	Household respondent aged 16+	United States	American Housing Survey	Secondary	3	Self-reported	Dichotomous (Y/N)	*Housing stability (3)*: Missed mortgage payments (homeowners); Missed rent payments (renters); Missed utility payments (all households)	*High* (5/5). Nationally representative AHS data. Disability classification validated against NHIS. Confounders controlled. Minimal missing data.

a While the housing quality scale was initially developed from low-income families with children, it was later proven to be reliable and valid among older population (Evans et al., 2002, 2008).

*Note.* AHS = American Housing Survey; CFA = confirmatory factor analysis; EU-SILC = European Union Statistics on Income and Living Conditions; HUD = U.S. Department of Housing and Urban Development; ICD-10 = International Classification of Diseases, 10th Revision; IQR = interquartile range; M = mean; MMAT = Mixed Methods Appraisal Tool; NHIS = National Health Interview Survey; N/R = not reported; SD = standard deviation; SIPP = Survey of Income and Program Participation; WHO = World Health Organization; Y/N = yes/no.

The included studies were predominantly conducted in the US (*n* = 9) and relied mainly on secondary analysis of large-scale survey databases (*n* = 10), with only three studies collecting primary data (Caffaro et al., 2016; Evans et al., 2000; Kim & Burgard, 2022). Sample sizes ranged from 207 participants to approximately 87.3 million hospitalization records. Seven studies specifically focused on older adults, though their age thresholds varied (60+, 62+, or 65+). The remaining six studies included broader age ranges and were not exclusively focused on older adult populations. The information source was predominantly self-report (*n* = 10), with one study relying solely on observer-rated assessment (Evans et al., 2000) and two using a combination of both (Jones-Rounds et al., 2014; Paredes et al., 2024).

### Quality appraisal results

Quality appraisal results are summarized in Table 2, with item-level ratings and supporting rationale provided in the Supplementary Material (MMAT Quality Assessment). All 13 included studies reached an acceptable level of methodological quality and met the quality threshold for inclusion: 5 were rated High, 5 Moderate-High, and 3 Moderate. The most frequently uncertain criterion concerned nonresponse bias, which was commonly rated “Can’t tell” in studies relying on secondary data, typically because the original data did not report nonresponse rates. The moderate ratings reflected limitations inherent to secondary data analysis rather than avoidable design choices (Canterberry et al., 2022; Rollings et al., 2022).

### Measurement tools

The 13 included studies used different housing insecurity tools. Because most of the studies relied on secondary data, they drew on items already available in large-scale surveys and constructed composite indicators tailored to specific research objectives, rather than developing instruments explicitly designed to measure housing insecurity. Only two primary studies developed or validated purpose-built tools: Caffaro et al. (2016) developed the self-reported Perceived Housing Quality Scale, and Evans et al. (2000) used an observer-rated Housing Quality Rating Scale.

Across the included studies, the number of items ranged from three to 45. Binary response formats were the most commonly used scoring approach, adopted by eight studies, while Likert-type scales were used in three studies and mixed or categorical formats in the remaining two.

### Dimension structures

**Figure 2:**
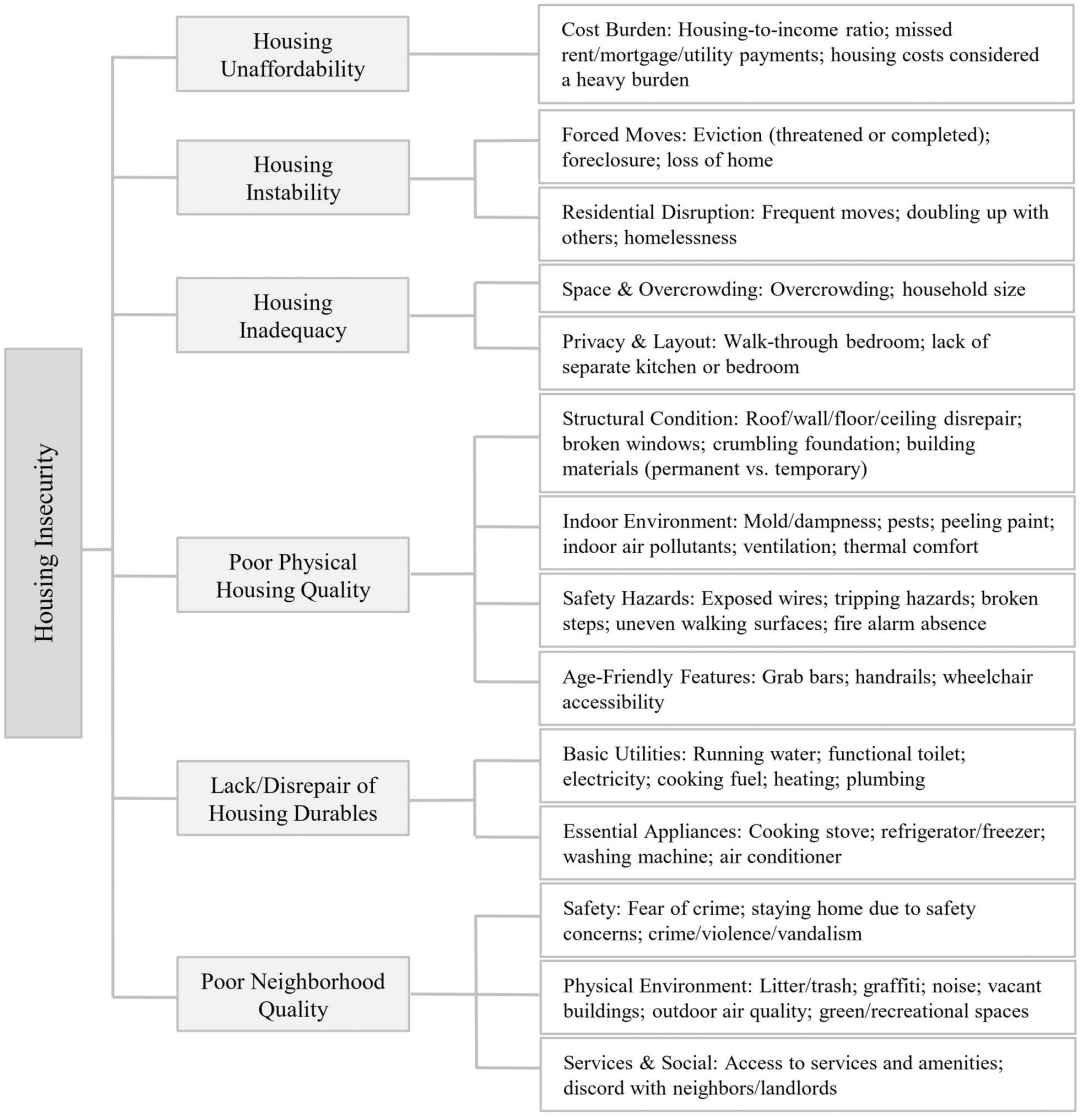
Included studies among older adults.

The detailed dimensions and their corresponding items are presented in Table 2, with variations in both the dimensions assessed and the number of items used across studies. Figure 2 synthesizes all dimensions and summarizes a conceptual map of housing insecurity dimensions and indicators identified across the included studies. Six core dimensions were identified: housing unaffordability, housing instability, housing inadequacy, poor physical housing quality, lack or disrepair of housing durables, and poor neighborhood quality. Poor physical housing quality was the most frequently addressed dimension, appearing across all 13 studies. Poor neighborhood quality was assessed in seven studies, and both housing unaffordability and housing instability were each captured in six studies. Housing inadequacy and lack or disrepair of housing durables were the least commonly measured dimensions: housing inadequacy-related indicators appeared in only three studies (Cheng et al., 2025; Evans et al., 2000; Kantz et al., 2023), and lack or disrepair of housing durables was more comprehensively measured only in Nolan and Winston (2011), with partial coverage in Cheng et al. (2025).

Considerable variation was evident in the scope and boundary of indicators selected across studies. For poor physical housing quality, most studies focused on structural conditions and indoor environment, while safety/hazard indicators appeared in fewer studies. Notably, Evans et al. (2000) was the only study to address age-friendly housing characteristics, including mobility support features such as hallway grab bars, pointing to a systematic gap in the existing literature. Housing unaffordability was operationalized either through housing-cost-to-income ratios or through specific payment difficulty events, and housing instability was measured through events ranging from frequent moves to eviction and homelessness. Housing inadequacy and lack or disrepair of housing durables were typically measured through summed indicators of basic utilities and facilities. Neighborhood quality indicators commonly covered perceived safety and physical maintenance, with Jones-Rounds et al. (2014) employing the most comprehensive approach by combining resident self-ratings with observer-rated assessments.

Regarding factor structure, only two of the 13 studies conducted formal factor analyses. Caffaro et al. (2016) confirmed a two-factor structure for the Perceived Housing Quality Scale through confirmatory factor analysis with satisfactory model fit, and Evans et al. (2000) extracted six factors through principal component analysis, collectively explaining 32% of total variance. The remaining 11 studies conducted no factor analysis, instead summing items directly into composite or subscale scores without examining the interrelationships among dimensions.

### Psychometric properties of measurement tools

As summarized in Table 3, reporting of reliability and validity was sparse across the 13 included studies. For internal consistency, only two studies provided data: Evans et al. (2000) reported a Cronbach’s alpha of 0.78 for their observers-rated scale, and Bhat et al. (2022) reported a coefficient of 0.69 for their housing insecurity index. Evans et al. (2000) was the only study to report interobserver reliability (Ebel *r* = 0.72), and no study reported test-retest reliability, leaving the temporal stability of all included instruments unexamined. For validity, seven studies provided some form of evidence while six provided none. Content validity was most referenced, primarily through anchoring indicators to HUD standards or existing literature. Construct validity evidence was limited to two primary data studies: Caffaro et al. (2016) drew on confirmatory factor analysis and correlations with external variables, while Evans et al. (2000) used factor analysis, discriminate validity checks, and pre-post relocation comparisons. Notably, no study reported concurrent or predictive validity, leaving associations between these instruments and other validated measures or health outcomes largely unestablished.

**Table 3:** Psychometric properties of housing insecurity measures in older adults.

Article authors	Internal consistency (*α*)	Other reliability	Validity reported (Y/N)	Validity type and evidence
Bhat et al. (2022)	0.69 (95% CI: 0.67, 0.71)	N/R	Y	N/R
Caffaro et al. (2016)	N/R	N/R	Y	*Content validity*: discussion with psychologists and architects *Construct validity*: Confirmatory factor analysis confirmed bidimensional structure (Comparative Fit Index = .916, Tucker-Lewis Index = .900, Root Mean Square Error of Approximation = .061, suggesting good model fit); association with other variables (b = ‒.17, *p* < .001)
Canterberry et al. (2022)	N/R	N/R	Y	N/R
Cheng et al. (2025)	N/R	N/R	Y	*Content validity*: indicators based on literature and clinical expertise
Evans et al. (2000)	0.78	Interobserver reliability: Ebel *r *= 0.72	Y	*Construct validity*: principal component factor analysis (account for 32% of total variance, significant correlations between subscales); test the discriminant ability by comparing with houses with known quality; use the scale in pre- and post-relocation sample
Golant and LaGreca (1994)	N/R	N/R	Y	*Content validity*: measures based on Department of Housing and Urban Development definition and literature *Construct validity*: significant correlation between indicators (Pearson *r* ranged from 0.13 to 0.83)
Jones-Rounds et al. (2014)	N/R	N/R	N	N/R
Kantz et al. (2023)	N/R	N/R	N	N/R
Kim and Burgard (2022)	N/R	N/R	N	N/R
Nolan and Winston (2011)	N/R	N/R	N	N/R
Paredes et al. (2024)	N/R	N/R	Y	*Content validity*: measures based on Department of Housing and Urban Development definition and literature
Rollings et al. (2022)	N/R	N/R	N	N/R
Trivedi et al. (2025)	N/R	N/R	N	N/R

*Note.* N/R, not reported.

## Discussion

This review systematically examined measurement tools for housing insecurity among older adults, evaluating 13 eligible studies with respect to tool form, dimensional coverage, and psychometric properties. The findings suggest several concerning patterns across the reviewed studies: considerable fragmentation in measurement approaches, uneven dimensional coverage, the near-universal absence of age-friendly indicators, and insufficient reporting of psychometric properties. These features may limit the comparability and policy relevance of existing research on housing insecurity and aging.

No included study sought to develop a standardized housing insecurity scale designed specifically for older adults. Most studies drew indicators from secondary surveys and constructed composite indices tailored to specific research objectives, rather than developing purpose-built and validated instruments (Cox et al., 2019; Leopold et al., 2016). This stands in notable contrast to the fields of food insecurity and water insecurity, where standardized, cross-culturally validated tools have been established and have provided a reliable basis for policy monitoring and intervention evaluation (Coates et al., 2007; Young et al., 2019). While secondary surveys offer analytical efficiency and large samples, their items were not designed to measure housing insecurity, and the selection of indicators is bounded by existing data structures, making it difficult to incorporate the measurement dimensions most relevant to older adults’ distinctive circumstances (Cox et al., 2017; Routhier, 2019). However, when developing a housing insecurity index for the adult population using the American Housing Survey, Murdoch et al. (2022) demonstrated that a transferable, psychometrically rigorous housing insecurity index can be constructed from existing survey data through confirmatory factor analysis and latent profile analysis, offering a methodological template that future aging-focused housing insecurity scale development could build upon. Although three included studies collected primary data, none targeted older adults, again highlighting the need for an age‑specific housing insecurity measure.

The inconsistent dimension structure and lack of context-specific components were evident in the dimensions assessed. Specifically, studies employed between one and seven dimensions to measure housing insecurity, reflecting limited consensus on its multidimensional structure and complicating tool development (Cox et al., 2017; Routhier, 2019). Synthesizing the dimensions identified across the 13 reviewed studies, our six‑dimensional framework offers a starting point for conceptualizing measurement among older adults (Wahl et al., 2012). It is also worth noting that most of these scales were developed within the US or Europe context, and their dimensional weights reflect the housing risk distribution specific to that social policy environment. In low- and middle-income country settings, access to basic utilities and physical housing conditions may carry greater relative importance (Lu et al., 2025). In highly urbanized and densely populated Asian societies, housing affordability and residential adequacy may warrant greater emphasis (Helble et al., 2021; King et al., 2017). In cultural contexts that place strong emphasis on community ties, the social dimensions of the residential environment (Yen et al., 2009) also merit more systematic measurement attention, aspects that have received limited attention in existing literature.

These differences suggest that housing insecurity measurement tools cannot be straightforwardly transferred across social and cultural contexts but rather require local adaptation, including the localization of item wording, context-sensitive calibration of dimensional weights, and revalidation within target populations to reflect local economic conditions, infrastructure, language practices, climatic and geographic characteristics, and cultural meanings of home (Sidani et al., 2010; Sousa & Rojjanasrirat, 2011). Instruments without adequate adaptation may encounter measurement equivalence issues when applied across diverse populations, limiting the cross-contextual comparability of findings (Byrne & van de Vijver, 2010). These observations point to an important direction for future tool development. Future efforts to develop housing insecurity measurement tools for older adults should therefore incorporate local adaptation and cross-cultural validity testing as standard components of the development process.

The near-universal absence of age-friendly housing indicators is the most consequential finding of the dimensional analysis. Among all 13 included studies, only Evans et al. (2000) incorporated mobility support features such as hallway grab bars within their observer-rated rating scale; no other study addressed any age-friendly housing characteristics. As physical and cognitive capacity may change with age, features including grab bars, accessible pathways, bathroom safety modifications, and fall prevention elements carry direct implications for older adults’ residential safety and capacity for independent daily living (Iwarsson & Ståhl, 2003; Wahl et al., 2012; WHO, 2018). The WHO Housing and Health Guidelines (2018) explicitly identify functional housing suitability as a core assessment dimension distinct from physical material quality, particularly for individuals with functional impairments, yet existing measurements in this review have largely failed to operationalize this emphasis. The reviewed tools appear limited in their capacity to fully characterize older adults’ housing vulnerability and to adequately support the design and evaluation of housing policies specifically targeting this population. These findings point to a clear gap that future measurement frameworks should seek to address.

From the perspective of environmental gerontology, older adults’ daily lives are highly concentrated within the home and neighborhood environment, and the influence of housing conditions on their functional independence and health deepens with age (Oswald et al., 2011; Wahl et al., 2012), providing theoretical grounding for extending housing insecurity measurement beyond general physical quality to encompass a broader range of dimensions. Building on this, Person-Environment Fit theory offers a more precise mechanistic account of why age-friendly dimensions carry particular conceptual importance in housing insecurity measurement for older adults: as health declines with age, the match between housing environment and individual capacity becomes increasingly consequential for health outcomes, and housing deficiencies may have more pronounced health impacts on older adults than younger populations (Lawton & Nahemow, 1973; Park et al., 2017; Wahl & Gerstorf, 2020). From this perspective, measurement tools that fail to incorporate age-friendly physical features may conceptually overlook the key age-specific dimensions of older adults’ residential environment, thereby limiting their ability to capture the residential risks associated with person-environment mismatch (Iwarsson & Ståhl, 2003).

Additionally, the predominance of self-report as the information source also carries particular methodological limitations in older adult populations. Self-report may face accuracy challenges in the context of cognitive decline, and certain objective housing deficiencies, such as structural safety hazards, may not be reliably detectable through residents’ subjective perceptions (Chaudhury & Oswald, 2019; Magaziner et al., 1988). Evans et al. (2000), the only study to rely exclusively on trained observer assessment, provided the most complete psychometric information of any study in this review. Mixed-source designs combining resident self-report with independent observer assessment represent an important methodological reference point (Evans et al., 2000; Jones-Rounds et al., 2014). Building on this finding, we suggest that a unified rating scale would be valuable to facilitate the tool development and enable cross-study comparison (Boateng & Adams, 2023; Murdoch et al., 2022).

Regarding factor structure, only two studies conducted formal factor analyses; the remaining studies summed items directly into composite or subscale scores without examining the interrelationships among dimensions. This leaves open the question of whether the dimensions of housing insecurity form an identifiable higher-order construct, and the assumption of dimensional independence lacks empirical support (Cox et al., 2017). Boateng and Adams (2023) demonstrated the viability of a higher-order factor structure for housing insecurity in slums and informal settlements through second-order confirmatory factor analysis, showing that multiple dimensions can be integrated within a unified overarching construct. These findings suggest that this methodological approach merits broader adoption in housing insecurity for older adults.

The limited and incomplete examination on psychometric properties mirrors findings from other measurement tool reviews. For example, the systematic evaluation of ageism scales and resilience measurement for children identified systematic underreporting of reliability and validity (Ayalon et al., 2019; Hall et al., 2024), suggesting a pervasive problem in social science scale development. In our review, internal consistency data was reported in only two studies, interobserver reliability in only one, and test-retest reliability was not reported in any study. Validity evidence was primarily confined to informal content validity justifications grounded in reference to existing literature or policy standards. Without concurrent or predictive validity testing, the associations between these tools and validated health outcomes remain unclear. This situation makes it difficult for researchers to make evidence-based judgments in tool selection and limits the comparability and synthesis of findings across studies (Mokkink et al., 2010; Terwee et al., 2007).

### Limitations

This review has several limitations. The restriction to English-language publications may under-represent the diversity of housing insecurity measurement practices in non-English-speaking contexts. With respect to eligibility criteria, the requirement for explicit reporting of scale development or psychometric evaluation means that studies employing housing-related indicators without framing them as scale development research were not captured, which may affect the representativeness of conclusions. With respect to the search strategy, the measurement-focused keyword design may not have captured all studies that employed a quantitative housing insecurity measure, though a sensitivity check indicated that this did not materially affect the final inclusion set (see Supplementary Material). Additionally, the MMAT appraisal results reflect the overall methodological quality of the included studies rather than the psychometric rigor of the measurement tools they employed. The relatively small number of included studies and their heavy concentration in the US or Western countries also constrain the robustness of cross-cultural conclusions, and the transferability of findings to other social policy and housing market contexts warrants further examination. The exclusion of gray literature is also a limitation. Nevertheless, included studies such as Golant and LaGreca (1994) constructed their measures based on government-administered surveys and HUD-defined housing standards, suggesting that key policy-relevant tools were represented in the peer-reviewed literature. Future reviews may benefit from incorporating gray literature to further enhance coverage.

### Implications

These findings carry important implications for both research methods and policy practice. At the research level, there is a clear need for standardized housing insecurity scales designed specifically for older adult populations. Synthesizing the dimensions identified across the 13 reviewed studies, a six-dimensional conceptual map incorporating housing unaffordability, instability, physical quality, adequacy, durables, and neighborhood quality, alongside the systematic integration of age-friendly housing characteristics, may serve as a useful starting point for future tool development. Tool development should incorporate context‑specific characteristics, tailoring items to local conditions and weighting dimensions according to economic, geographic, sociocultural, and policy contexts. Such tools should undergo comprehensive psychometric validation prior to deployment, including comprehensive tests of reliability and validity. With respect to reliability, internal consistency (Cronbach’s *α*) should be reported as a standard requirement (Terwee et al., 2007), test-retest reliability should be established as a necessary indicator of temporal stability, and interrater reliability should be documented for instruments employing multiple raters (Mokkink et al., 2010). With respect to validity, structural validity should be examined through confirmatory factor analysis rather than direct item summation; content validity should be grounded in consultation with target populations and domain experts rather than informal literature references; and the assessment of concurrent and predictive validity warrants greater attention to establish how tool scores relate to other validated measures and relevant health outcomes (Boateng et al., 2018). Precedents from adjacent fields, including the Household Food Insecurity Access Scale (Coates et al., 2007) and the Household Water InSecurity Experiences Scale (Young et al., 2019), offer useful methodological reference points, as both underwent rigorous development pathways encompassing comprehensive content validation and psychometric testing. Mixed-source designs combining resident self-report with professional observer assessment are recommended, as they allow for the independent verification of objective housing conditions alongside subjective resident perceptions, which is particularly valuable when cognitive limitations may affect self-reporting accuracy. At the policy level, the establishment of standardized tools will provide an essential foundation for tracking progress on policy goals in different societal contexts. These recommendations are summarized in Table 4, which outlines the key gaps identified in this review, corresponding recommendations for future tool development, and the rationale underpinning each recommendation.

**Table 4:** Recommendations for developing housing insecurity measurement among older adults.

Category	What (identified gap)	How (recommended action)	Why (rationale)
Tool design	Lack of purpose-built tool specifically for older adults; reliance on items from secondary survey	Develop primary instruments through systematic item development involving older adults and domain experts	Secondary survey items constrain dimensional coverage and preclude rigorous psychometric evaluation.
Dimensional coverage (1)	Absence of age-friendly features (e.g., grab bars, accessible pathways, fall prevention)	Incorporate age-friendly physical features as an independent dimension, informed by WHO Housing and Health Guidelines and Person-Environment Fit theory	As individuals age, housing-environment fit becomes increasingly consequential for functional independence and health outcomes among older adults
Dimensional coverage (2)	Uneven coverage across six core dimensions; underrepresentation of housing inadequacy and durables	Adopt the six-dimensional framework synthesized from the reviewed studies (unaffordability, instability, physical quality, inadequacy, durables, neighborhood quality) as a reference structure	Uneven coverage limits the comprehensiveness of measurement tool and reduces cross-study comparability.
Information source	Predominates use of Self-report items; limited capture of objective deficiencies (e.g., structural hazards)	Adopt mixed-source designs combining self-report with trained observer assessment; develop unified observer rating protocols	Mixed-source designs improve objectivity and reliability of the tool.
Reliability	Inadequate assessment and reporting of reliability	Report Cronbach’s alpha as standard to indicate internal consistency reliability; establish test-retest reliability for temporal stability; document interrater reliability for observer-rated instruments	Without reliability evidence, the consistency of tools cannot be evaluated, limiting their utility in longitudinal research and policy monitoring.
Validity	Limited and informal validity evidence; absence of concurrent or predictive validity	Examine structural validity via confirmatory factor analysis; establish content validity through expert and population consultation; assess concurrent and predictive validity formally	Without validity evidence, the meaningfulness of tool scores cannot be confirmed, impeding instrument selection and cross-study synthesis.
Factor structure	Direct summation without examining dimensional interrelationships; untested dimensional independence	Conduct confirmatory factor analysis and explore higher-order structures to test whether dimensions form a unified overarching construct	Sound factor structure is essential for justified composite scoring and theoretical coherence in housing insecurity measurement.
Cross-cultural applicability	Concentration of tools in the US or European contexts; limited cultural and policy transferability dimensional weights	Incorporate systematic cultural adaptation and cross-cultural validity testing as standard components of tool development	Without adaptation, measurement equivalence cannot be assumed across diverse populations, limiting cross-contextual comparability and policy relevance.

*Note.* Recommendations are synthesized from findings across the 13 reviewed studies and informed by environmental gerontology and person-environment fit theory.

## Conclusion

This review is the first to systematically examine measurement tools for housing insecurity among older adults. The existing literature lacks a standardized scale designed for this group, and current measurement practice is characterized by tool fragmentation, uneven dimensional coverage, and insufficient psychometric reporting. At the dimensional level, age-friendly housing characteristics were largely absent from the reviewed tools, while housing adequacy and durables appeared to be relatively undermeasured, suggesting that existing measurement frameworks may not fully capture the scope of older adults’ housing vulnerability. At the psychometric level, internal consistency, test-retest reliability, and concurrent validity were rarely reported across the reviewed studies, which may constrain the evaluability of available tools. These findings collectively point to a core priority: the field requires purpose-built, systematically validated instruments that incorporate age-friendly dimensions, and future research should advance the local adaptation and validation of such tools across diverse policy and housing settings, with the ultimate aim of providing a reliable and valid measurement foundation for evidence-based housing policy for older adults.

## Supplementary Material

gnag130_Supplementary_Data

## Data Availability

This systematic review was conducted in accordance with the Preferred Reporting Items for Systematic Reviews (PRISMA 2020) checklist. The review protocol was pre-registered on PROSPERO (CRD420251230421; available at https://www.crd.york.ac.uk/PROSPERO/view/CRD420251230421). This systematic review synthesized data from published primary studies. All included studies and their citations are provided in the reference list and Supplementary Material. The data extraction forms and coding frameworks developed for this review are available upon reasonable request.
