# Supplementary material for: How is housing insecurity measured among older adults? A systematic review
Source: Gerontologist. 2026 Jun 12;66(7):gnag130. doi: 10.1093/geront/gnag130 (PMC13335642; doi:10.1093/geront/gnag130)
Supplement: gnag130_Supplementary_Data [file gnag130_supplementary_data.pdf]

Supplementary Materials

How is housing insecurity measured among older adults? A systematic review

Tianxin Cai, Peng Cheng, Sin Yu Lam, Peiyi Lu

Contents

Sensitivity Check for Search Strategy

Table S1. Complete database-specific search strings

Mixed Methods Appraisal Tool (MMAT) Quality Assessment

Sensitivity Check for Search Strategy

To evaluate the impact of the measurement-focused keyword group (Group 2) on literature coverage, a sensitivity check was conducted in PsycINFO. The broader search retained Group 1 (housing insecurity-related terms) and Group 3 (older adult-related terms) while removing Group 2 (measurement-related terms). The simplified search retrieved 404 records, compared with 166 records yielded by the original three-block search, resulting in 238 additional records.

To draw a sample from the 404 records, systematic random sampling was employed. A random number generator was used to determine the starting point, which was randomly set at record 3. Every fourth record was then selected (i.e., records 3, 7, 11, 15...), yielding a sample of 100 records. Titles and abstracts of the 100 sampled records were screened against eligibility criterion, which required that studies employed a quantitative measure of housing insecurity. None of the 100 sampled records met this criterion, indicating that the additional records retrieved by the broader search did not employ quantitative housing insecurity measures and would have been excluded at the screening stage regardless of retrieval. This finding suggests that the measurement-focused search design did not materially affect the final inclusion set.

Table S1 Complete database-specific search strings

**PsycInfo**
tiab(("housing instabilit*" OR "housing insecurit*" OR "residential insecurit*" OR "residential instabilit*" OR "housing povert*" OR "housing vulnerab*" OR "housing affordab*" OR "housing cost burden*" OR "rent burden*" OR "housing tenure" OR "housing precarit*" OR "residential precarit*" OR "housing stress" OR "housing quality") AND (measure* OR scale* OR instrument* OR assessment* OR indicator* OR questionnaire* OR tool* OR index OR survey* OR validat* OR reliab* OR adapt* OR psychometric*) AND (aged OR elder* OR senior* OR "older adult*" OR "older person*" OR "older people" OR ag?ing OR "old age" OR geriatric* OR gerontolog* OR "late life") NOT ("animals" NOT ("animals" AND "humans")))
**PubMed**
(("housing instabilit*"[MeSH Terms] OR "housing insecurit*"[Title/Abstract] OR "residential insecurit*"[Title/Abstract] OR "residential instabilit*"[Title/Abstract] OR "housing povert*"[Title/Abstract] OR "housing vulnerab*"[Title/Abstract] OR "housing affordab*"[Title/Abstract] OR "housing cost burden*"[Title/Abstract] OR "rent burden*"[Title/Abstract] OR "housing tenure"[Title/Abstract] OR "housing precarit*"[Title/Abstract] OR "residential precarit*"[Title/Abstract] OR "housing stress"[Title/Abstract] OR "housing quality"[Title/Abstract]))<br>AND<br>(("Surveys and Questionnaires"[MeSH] OR "Psychometrics"[MeSH] OR "Reproducibility of Results"[MeSH])<br>OR<br>(measure*[Title/Abstract] OR scale*[Title/Abstract] OR index[Title/Abstract] OR instrument*[Title/Abstract] OR assessment*[Title/Abstract] OR indicator*[Title/Abstract] OR questionnaire*[Title/Abstract] OR tool*[Title/Abstract] OR survey*[Title/Abstract] OR validat*[Title/Abstract] OR reliab*[Title/Abstract] OR adapt*[Title/Abstract] OR psychometric*[Title/Abstract]))<br>AND<br>((aged[MeSH Terms])<br>OR<br>(elder*[Title/Abstract] OR senior*[Title/Abstract] OR "older adult*"[Title/Abstract] OR "older person*"[Title/Abstract] OR "older people"[Title/Abstract] OR ag?ing[Title/Abstract] OR "old age"[Title/Abstract] OR geriatric*[Title/Abstract] OR gerontolog*[Title/Abstract] OR "late life"[Title/Abstract]))<br>NOT ("Animals"[MeSH] NOT "Humans"[MeSH])
**Web of Science**<br>(TI=("housing instabilit*" OR "housing insecurit*" OR "residential insecurit*" OR "residential instabilit*" OR "housing povert*" OR "housing vulnerab*" OR "housing affordab*" OR "housing cost burden*" OR "rent burden*" OR "housing tenure" OR "housing precarit*" OR "residential precarit*" OR "housing stress" OR "housing quality")<br>OR<br>AB=("housing instabilit*" OR "housing insecurit*" OR "residential insecurit*" OR "residential instabilit*" OR "housing povert*" OR "housing vulnerab*" OR "housing affordab*" OR "housing cost burden*" OR "rent burden*" OR "housing tenure" OR "housing precarit*" OR "residential precarit*" OR "housing stress" OR "housing quality"))<br>AND<br>(TI=(measure* OR scale* OR index OR instrument* OR assessment* OR indicator* OR questionnaire* OR tool* OR survey* OR validat* OR reliab* OR adapt* OR psychometric*)<br>OR<br>AB=(measure* OR scale* OR index OR instrument* OR assessment* OR indicator* OR questionnaire* OR tool* OR

survey* OR validat* OR reliab* OR adapt* OR psychometric*))<br>AND<br>(TI=(elder* OR senior* OR "older adult*" OR "older person*" OR "older people" OR ag?ing OR "old age" OR geriatric* OR gerontolog* OR "late life")<br>OR<br>AB=(elder* OR senior* OR "older adult*" OR "older person*" OR "older people" OR ag?ing OR "old age" OR geriatric* OR gerontolog* OR "late life"))<br>NOT<br>(TS=(animal* OR rat OR rats OR mouse OR mice) NOT TS=(human* OR patient*))
**Sociological Abstracts**
title(("housing instabilit*" OR "housing insecurit*" OR "residential insecurit*" OR "residential instabilit*" OR "housing povert*" OR "housing vulnerab*" OR "housing affordab*" OR "housing cost burden*" OR "rent burden*" OR "housing tenure" OR "housing precarit*" OR "residential precarit*" OR "housing stress" OR "housing quality") AND (measure* OR scale* OR instrument* OR assessment* OR indicator* OR questionnaire* OR tool* OR index OR survey* OR validat* OR reliab* OR adapt* OR psychometric*) AND (aged OR elder* OR senior* OR "older adult*" OR "older person*" OR "older people" OR ag?ing OR "old age" OR geriatric* OR gerontolog* OR "late life") NOT ("animals" NOT ("animals" AND "humans"))) OR abstract(("housing instabilit*" OR "housing insecurit*" OR "residential insecurit*" OR "residential instabilit*" OR "housing povert*" OR "housing vulnerab*" OR "housing affordab*" OR "housing cost burden*" OR "rent burden*" OR "housing tenure" OR "housing precarit*" OR "residential precarit*" OR "housing stress" OR "housing quality") AND (measure* OR scale* OR instrument* OR assessment* OR indicator* OR questionnaire* OR tool* OR index OR survey* OR validat* OR reliab* OR adapt* OR psychometric*) AND (aged OR elder* OR senior* OR "older adult*" OR "older person*" OR "older people" OR ag?ing OR "old age" OR geriatric* OR gerontolog* OR "late life") NOT ("animals" NOT ("animals" AND "humans")))
**Scopus**
((TITLE("housing instabilit*" OR "housing insecurit*" OR "residential insecurit*" OR "residential instabilit*" OR "housing povert*" OR "housing vulnerab*" OR "housing affordab*" OR "housing cost burden*" OR "rent burden*" OR "housing tenure" OR "housing precarit*" OR "residential precarit*" OR "housing stress" OR "housing quality") OR ABS("housing instabilit*" OR "housing insecurit*" OR "residential insecurit*" OR "residential instabilit*" OR "housing povert*" OR "housing vulnerab*" OR "housing affordab*" OR "housing cost burden*" OR "rent burden*" OR "housing tenure" OR "housing precarit*" OR "residential precarit*" OR "housing stress" OR "housing quality")) AND (TITLE(measure* OR scale* OR instrument* OR assessment* OR indicator* OR questionnaire* OR tool* OR index OR survey* OR validat* OR reliab* OR adapt* OR psychometric*) OR ABS(measure* OR scale* OR instrument* OR assessment* OR indicator* OR questionnaire* OR tool* OR index OR survey* OR validat* OR reliab* OR adapt* OR psychometric*)) AND (TITLE(aged OR elder* OR senior* OR "older adult*" OR "older person*" OR "older people" OR ag?ing OR "old age" OR geriatric* OR gerontolog* OR "late life") OR ABS(aged OR elder* OR senior* OR "older adult*" OR "older person*" OR "older people" OR ag?ing OR "old age" OR geriatric* OR gerontolog* OR "late life")) AND NOT (TITLE-ABS-KEY(animals) AND NOT TITLE-ABS-KEY(animals AND humans))) AND (LIMIT-TO (LANGUAGE,"English")) AND (LIMIT-TO (DOCTYPE,"ar") OR LIMIT-TO (DOCTYPE,"re"))

Mixed Methods Appraisal Tool (MMAT) Quality Assessment

Each criterion was rated as Yes, No, or Can’t tell in accordance with the MMAT 2018 guidelines. Prior to criterion-level appraisal, two screening questions (S1: Are there clear research questions? S2: Do the collected data allow to address the research questions?) were applied; all 13 included studies met both screening criteria and proceeded to full appraisal. Overall methodological quality was judged holistically based on the pattern of ratings across the five criteria: studies with all five criteria rated Yes were classified as High quality; four Yes ratings as Moderate–High; three Yes ratings (including those with one or more Can’t tell responses) as Moderate; and studies with two or fewer Yes ratings or any No rating were classified as Moderate or below.

Study	Study Type (MMAT)	S1	S2	Criterion	Criterion Description	Rating	Rationale / Evidence from Article	Overall Quality	Inclusion Decision
**Caffaro et al. (2016)**	Quantitative Descriptive (Type 4)	Yes	Yes	4.1	Is the sampling strategy relevant to address the research question?	**Yes**	Quota sampling by gender (N=285 Italian adults); proportional to Italian national population distribution. Strategy and rationale clearly described.	**High**	**Included**
				4.2	Is the sample representative of the target population?	**Yes**	Gender-quota sampling matched Italian national demographic data; sample description provided (age M=42.81, SD=12.73).		
				4.3	Are the measurements appropriate?	**Yes**	Content validity via discussion with psychologists and architects; construct validity confirmed by CFA (CFI=.916, TLI=.900, RMSEA=.061). 11-item, 7-point Likert scale, two-factor structure.		
				4.4	Is the risk of nonresponse bias low?	**Can't tell**	Snowball recruitment; non-response rate not reported; no comparison of respondents vs. non-respondents.		
				4.5	Is the statistical analysis appropriate?	**Yes**	CFA used to confirm bidimensional structure; correlations with external variables (b=−.17, p<.001) reported as construct validity evidence.		
**Kantz et al. (2023)**	Quantitative Non-Randomized (Type 3)	Yes	Yes	3.1	Are the participants representative of the target population?	**Yes**	Secondary analysis of SIPP (U.S. Census Bureau multi-stage stratified probability sample). N=10,858 adults aged 62–87 (M=72.0). Nationally representative.	**High**	**Included**
				3.2	Are measurements appropriate?	**Yes**	Housing quality (5 items), affordability (2 items), stability (moves), and neighbourhood safety (2 items) operationalized with binary SIPP items; ADL/IADL and self-rated health are established measures.		
				3.3	Are there complete outcome data?	**Yes**	Four waves of panel data; 37,333 observations; attrition described; complete case analysis applied.		
				3.4	Are the confounders accounted for?	**Yes**	Stratified logistic regression controlled for age, gender, race/ethnicity, income, education, marital status, and other sociodemographic covariates. Temporal ordering maintained across waves.		
				3.5	Is the exposure/intervention consistent during the study period?	**Yes**	Identical structured questionnaire items used across all four SIPP waves, enabling valid longitudinal comparison.		
**Kim & Burgard (2022)**	Quantitative Non-Randomized (Type 3)	Yes	Yes	3.1	Are the participants representative of the target population?	**Yes**	Stratified random sample (MRRS; N=255, 510 observations). Represents low-to-moderate income renters in Detroit metro area. Follow-up retention >90% across all three waves.	**High**	**Included**
				3.2	Are measurements appropriate?	**Yes**	Housing instability: composite 14-item index (homelessness, eviction, frequent moves, cost-related moves, substandard conditions). Mental health: PHQ-4 (validated 4-item scale).		
				3.3	Are there complete outcome data?	**Yes**	Three panel waves; >90% retention; no significant selective attrition detected.		
				3.4	Are the confounders accounted for?	**Yes**	Propensity score weighting for sociodemographic confounders; fixed-effects approaches for longitudinal comparisons. Methodologically strongest study in the review.		
				3.5	Is the exposure/intervention	**Yes**	Housing instability measured retrospectively at each wave using		

Study	Study Type (MMAT)	S1	S2	Criterion	Criterion Description	Rating	Rationale / Evidence from Article	Overall Quality	Inclusion Decision
					consistent during the study period?		identical items; PHQ-4 administered consistently at all three time points.		
**Nolan & Winston (2011)**	Quantitative Descriptive (Type 4)	Yes	Yes	4.1	Is the sampling strategy relevant to address the research question?	**Yes**	EU-SILC data; two-stage sampling proportional to population density across Irish regions. Strategy clearly described and justified.	**High**	**Included**
				4.2	Is the sample representative of the target population?	**Yes**	N=2,879 households (head aged ≥65); demographics compared with Irish national statistics confirming representativeness.		
				4.3	Are the measurements appropriate?	**Yes**	Four housing deprivation dimensions with binary items: housing quality (7 items), household durables (19 items), affordability (3 items), neighbourhood conditions (4 items). Clear conceptual justification for each dimension.		
				4.4	Is the risk of nonresponse bias low?	**Can't tell**	EU-SILC non-response rate not reported; no analysis comparing respondents vs. non-respondents.		
				4.5	Is the statistical analysis appropriate?	**Yes**	OLS regression models with robustness checks. Appropriate for descriptive and associative research questions.		
**Paredes et al. (2024)**	Quantitative Descriptive (Type 4)	Yes	Yes	4.1	Is the sampling strategy relevant to address the research question?	**Yes**	NHATS 2011; probability-based national random sample with oversampling of Black and age 90+ participants. Weighted estimates represent ~29.84 million community-living older Americans.	**High**	**Included**
				4.2	Is the sample representative of the target population?	**Yes**	Analytical weights applied; final N=6,466; Supplemental Table S1 confirms minimal selection bias from excluded participants.		
				4.3	Are the measurements appropriate?	**Yes**	Three housing insecurity domains: affordability (cost burden %), quality (10 items: NHATS assessor checklists + self-report), neighbourhood quality (4 items). Grounded in HUD definitions.		
				4.4	Is the risk of nonresponse bias low?	**Yes**	Weighted response rate 71%; systematic comparison of included vs. excluded participants documented in Supplemental Table S1.		
				4.5	Is the statistical analysis appropriate?	**Yes**	Analytical sampling weights for nationally representative prevalence estimates; chi-square tests for subgroup comparisons. Appropriate for population-based descriptive study.		
**Trivedi et al. (2025)**	Quantitative Non-Randomized (Type 3)	Yes	Yes	3.1	Are the participants representative of the target population?	**Yes**	American Housing Survey (AHS) biennial nationally representative data; N=56,438 (2017) and N=54,967 (2021). Survey weights applied throughout.	**High**	**Included**
				3.2	Are measurements appropriate?	**Yes**	Housing stability: three binary outcomes (missed mortgage/rent/utility payments). Disability classification via AHS six-item battery mapped to LTSS/Non-LTSS/Non-disability groups, validated against NHIS ADL/IADL data.		
				3.3	Are there complete outcome data?	**Yes**	Missing disability data: 2.32% (2017), 1.70% (2021); logistic regression confirmed non-systematic missingness. No additional imputation needed given small fraction and AHS pre-imputed data.		
				3.4	Are the confounders accounted for?	**Yes**	Multivariate logistic regression adjusted for gender, age, race/ethnicity, marital status, education, citizenship, household size, years in house, housing cost-to-income ratio, census division, metropolitan status, and HUD subsidy. Mean VIF=1.218–1.222 (no multicollinearity).		
				3.5	Is the	**Yes**	Identical AHS questionnaire structure		

Study	Study Type (MMAT)	S1	S2	Criterion	Criterion Description	Rating	Rationale / Evidence from Article	Overall Quality	Inclusion Decision
					exposure/intervention consistent during the study period?		and disability items used in both 2017 and 2021 waves; housing stability indicators operationalized consistently.		
**Bhat et al. (2022)**	Quantitative Non-Randomized (Type 3)	Yes	Yes	3.1	Are the participants representative of the target population?	**Yes**	MIDUS national longitudinal study; N=2,598 including Milwaukee African American oversample. Two waves: MIDUS 2 (2004–2006) and MIDUS 3 (2013–2015/2016–2017). Attrition analysis: no significant differences by health, age, or race between retained and lost participants.	**High**	**Included**
				3.2	Are measurements appropriate?	**Yes**	Housing Insecurity Index (HII): 5 binary items summed (0–5), Cronbach's α=0.69 (95% CI: 0.67–0.71). Self-rated health: dichotomized 5-point scale. Chronic conditions: validated 15-category count.		
				3.3	Are there complete outcome data?	**Yes**	Attrition analysis from MIDUS 2 to MIDUS 3 showed no significant differences by health, age, or race between analytical and excluded participants (Supplemental Table 1).		
				3.4	Are the confounders accounted for?	**Yes**	Logistic and Poisson regression controlled for age, race, gender, education, household income, marital status. MIDUS 2 baseline health included as covariate. Interaction terms (age×HI; race×HI) tested separately.		
				3.5	Is the exposure/intervention consistent during the study period?	**Yes**	Housing insecurity collected retrospectively in MIDUS 3 covering the inter-wave period; health outcomes consistently measured at both waves using identical instruments.		
**Canterberry et al. (2022)**	Quantitative Non-Randomized (Type 3)	Yes	Yes	3.1	Are the participants representative of the target population?	**Can't tell**	Limited to Humana Medicare Advantage beneficiaries; survey response rate 24.5% (105,901/431,215). Non-response bias on HRSN status cannot be excluded; findings may not generalise to all Medicare populations.	**Moderate**	**Included**
				3.2	Are measurements appropriate?	**Yes**	HRSNs (housing insecurity, poor housing quality) measured via binary indicators adapted from CMS AHC HRSN Screening Tool. Acute care utilization captured objectively through linked claims data; avoidable events classified using validated algorithms (AHRQ Prevention Quality Indicators; NYU ED algorithm).		
				3.3	Are there complete outcome data?	**Yes**	Linked medical claims data covering full calendar year 2019; complete administrative data with no missing outcome records for the analytical sample.		
				3.4	Are the confounders accounted for?	**Yes**	Negative binomial regression adjusted for age, sex, race/ethnicity, disability status, dual Medicare–Medicaid eligibility, Elixhauser Comorbidity Index, and hospital referral region (HRR) fixed effects.		
				3.5	Is the exposure/intervention consistent during the study period?	**Can't tell**	HRSNs measured once (Oct 2019–Feb 2020) whereas outcomes cover Jan–Dec 2019; survey conducted largely after the outcome period, creating temporal inconsistency limiting causal inference.		
**Cheng et al. (2025)**	Quantitative Non-Randomized (Type 3)	Yes	Yes	3.1	Are the participants representative of the target population?	**Yes**	LASI Wave 1 (2017–2018): multi-stage stratified probability sample covering 35 Indian states/union territories. N=30,632 aged ≥60 from 23,329 households. Oversampling of metropolitan areas corrected via survey weights.	**Moderate–High**	**Included**
				3.2	Are measurements appropriate?	**Yes**	Housing quality index: 5 binary indicators (housing materials, sanitation, water, cooking fuel, electricity), summed 0–5. Related		

Study	Study Type (MMAT)	S1	S2	Criterion	Criterion Description	Rating	Rationale / Evidence from Article	Overall Quality	Inclusion Decision
							environmental factors: 4 binary indicators. Falls: structured self-report with 2-year recall. Covariates with <10% missing imputed via multiple imputation.		
				3.3	Are there complete outcome data?	**Yes**	From 30,727 eligible adults, 95 excluded for missing fall data; final N=30,632 (99.7% retention). All covariates <10% missing; multiple imputation applied.		
				3.4	Are the confounders accounted for?	**Yes**	Generalised linear mixed models (GLMMs) with household-level random effects; three-step sequential models adjusting for sociodemographic, health, and lifestyle factors. Multiple sensitivity analyses performed.		
				3.5	Is the exposure/intervention consistent during the study period?	**Can't tell**	Cross-sectional design (LASI Wave 1 only); housing quality and falls measured at same time point; temporal ordering cannot be confirmed. Housing quality assessed via basic survey indicators rather than a standardised validated instrument.		
**Golant & LaGreca (1994)**	Quantitative Descriptive (Type 4)	Yes	Yes	4.1	Is the sampling strategy relevant to address the research question?	**Yes**	1987 American Housing Survey (AHS) — national probability sample of 43,436 housing units administered by U.S. Bureau of the Census. Focus on 12,859 householders aged ≥60.	**High**	**Included**
				4.2	Is the sample representative of the target population?	**Yes**	Sample represents 26,318,348 elderly households (29% of total national AHS sample). Separate analyses for homeowners and renters. National representativeness established via AHS design.		
				4.3	Are the measurements appropriate?	**Yes**	Three complementary housing quality measures: (1) HUD 3-level classification (adequate/moderately deficient/severely deficient); (2) authors' NUMERICAL count of up to 26 physical deficiencies; (3) six sub-category NUMERICAL measures. Both main measures correlated (r=0.57–0.64, p<.001). Operationalization detailed in Appendices A and B.		
				4.4	Is the risk of nonresponse bias low?	**Can't tell**	1987 AHS non-response rate not reported; no comparison of respondents vs. non-respondents. Non-response bias cannot be assessed from the article.		
				4.5	Is the statistical analysis appropriate?	**Yes**	Sequential multiple regression (Models 1–6) progressively controlling for building age, length-of-residence interaction effects, and dwelling/demographic/location variables. R^2 comparisons across nested models used to assess unique variance explained by length of residence.		
**Evans et al. (2000)**	Quantitative Non-Randomized (Type 3)	Yes	Yes	3.1	Are the participants representative of the target population?	**Can't tell**	Two independent samples described: (1) Cross-sectional rural sample: N=207 low-to-middle-income women (upstate New York; income:needs ratio M=1.69, SD=1.27; predominantly White 97%). (2) Longitudinal urban sample: N=31 low-income women (income:needs ratio M=1.10, SD=0.82; predominantly African American 61%) assessed before and after Habitat for Humanity relocation. However, both samples were restricted to low-income women with at least one child living at home, limiting representativeness of the broader older adult population.	**Moderate–High**	**Included**
				3.2	Are measurements appropriate?	**Yes**	Housing quality: observer-based Housing Quality Instrument (HQI), 88 items across 6 subscales (α=.78; subscale α=.71–.89; inter-observer		

Study	Study Type (MMAT)	S1	S2	Criterion	Criterion Description	Rating	Rationale / Evidence from Article	Overall Quality	Inclusion Decision
							reliability Ebel r=.72). 81/88 items observer-rated. Psychological distress: PERI Demoralization Index (α=.91). Construct validity via factor analysis, discriminant validity tests, and pre-post relocation changes.		
				3.3	Are there complete outcome data?	**Yes**	Cross-sectional sample: complete data from N=207. Longitudinal sample: N=31 assessed at both pre-relocation (mean 4.5 months before) and post-relocation (mean 7.3 months after). No missing data issues reported.		
				3.4	Are the confounders accounted for?	**Yes**	Cross-sectional: income:needs ratio statistically controlled (r=−.39). Longitudinal: pre-relocation PERI scores as covariate, controlling for pre-existing mental health; income also controlled.		
				3.5	Is the exposure/intervention consistent during the study period?	**Yes**	Both samples assessed using the same HQI instrument by trained raters. Pre-relocation data collected mean 4.5 months before move; post-relocation data 7.3 months after, with consistent measurement protocol at both time points.		
**Jones-Rounds et al. (2014)**	Quantitative Non-Randomized (Type 3)	Yes	Yes	3.1	Are the participants representative of the target population?	**Yes**	WHO LARES data: N=5,605 European adults (18–64 years) from 8 cities. Random sampling proportional to city size (600–1,700 per city). City-level fixed effects included in models.	**Moderate**	**Included**
				3.2	Are measurements appropriate?	**Yes**	Housing quality: LARES rater assessment of visible disrepair in kitchen, bathroom, corridor, and bedroom — summed additive score. Neighbourhood quality: composite of resident self-ratings and LARES observer scores (green spaces, vegetation, graffiti, litter). Psychological well-being: 9-item validated scale (α=0.86). All raters received harmonised cross-city training.		
				3.3	Are there complete outcome data?	**Can't tell**	Income data had substantial missing values and could not be included in the SES composite. Overall response rate and missing data patterns for other variables not reported.		
				3.4	Are the confounders accounted for?	**Yes**	Multilevel random coefficient modelling for hierarchical data structure (individuals nested within neighbourhoods). Statistical controls: SES composite, gender, marital status, employment status; city as fixed effect.		
				3.5	Is the exposure/intervention consistent during the study period?	**Can't tell**	Cross-sectional design. Neighbourhood quality composite includes resident subjective ratings that may be influenced by current psychological well-being (shared methods variance). Temporal ordering between exposures and outcome cannot be established.		
**Rollings et al. (2022)**	Quantitative Non-Randomized (Type 3)	Yes	Yes	3.1	Are the participants representative of the target population?	**Yes**	National Inpatient Sample (NIS) 2017–2019: the largest all-payer inpatient database in the US (~85% of community hospitals). Weighted analysis of 87,348,604 hospitalisations provides nationally representative estimates for adults aged 18–99. Survey sampling weights applied.	**Moderate**	**Included (noted limitations)**
				3.2	Are measurements appropriate?	**Can't tell**	Housing instability operationalized via 5 ICD-10 Z59-codes. However, 96.8% of coded cases used Z59.0 (homelessness only); prior literature shows Z-codes appear in only 1–2% of inpatient records, suggesting significant underidentification of the broader housing instability continuum.		
				3.3	Are there complete outcome data?	**Yes**	Full administrative NIS data for 2017–2019; exclusions limited to records with incomplete study data (eTable 2).		

Study	Study Type (MMAT)	S1	S2	Criterion	Criterion Description	Rating	Rationale / Evidence from Article	Overall Quality	Inclusion Decision
							No missing outcome data for the analytical sample.		
				3.4	Are the confounders accounted for?	**No**	All comparisons are unadjusted (descriptive statistics and crude ORs only). No adjustment for age, sex, race/ethnicity, insurance type, or comorbidity burden. Authors explicitly acknowledge this as a primary limitation; causal inference is not supported.		
				3.5	Is the exposure/intervention consistent during the study period?	**Yes**	Both exposure (Z59-codes) and primary outcome (hospitalization diagnosis) derived from the same NIS record. ICD-10 coding applied uniformly across 2017–2019. Sensitivity analyses stratified by each of the 5 Z59-codes confirmed consistency of general trends.		
